# PRKN Ser131 phosphorylation promotes cigarette smoke-induced mitophagy impairment and epithelial cell senescence via MKK3/p38 MAPK activation: An in vitro and in vivo study

**DOI:** 10.18332/tid/218816

**Published:** 2026-05-27

**Authors:** Yajie Hu, Jianyu Liu, Mengyu Zhang, Jiajia Qu, Wenqiang Hao, Xinmiao Zhu, Dawei Wu, Shuo Jiang, Yiqing Qu

**Affiliations:** 1 Department of Pulmonary and Critical Care Medicine, Laboratory of Basic Medical Sciences, Qilu Hospital of Shandong University, Jinan, China; 2 Department of Respiratory and Critical Care Medicine, Zhuzhou Central Hospital, Zhuzhou, China

**Keywords:** chronic obstructive pulmonary disease, mitophagy, cellular senescence, MKK3, PRKN

## Abstract

**INTRODUCTION:**

Cigarette smoke (CS) exposure impairs mitochondrial function and promotes senescence in airway epithelial cells, contributing to the pathogenesis of chronic obstructive pulmonary disease (COPD). However, the molecular mechanisms linking mitophagy dysfunction to cellular senescence remain poorly understood. Parkin (PRKN) is a key regulator of mitophagy, but whether PRKN Ser131 phosphorylation contributes to CS-induced impairment of mitophagy and senescence remains unclear. Therefore, this study aimed to investigate the role of PRKN Ser131 phosphorylation in CS-induced impairment of mitophagy and epithelial cell senescence, and to explore the underlying mechanism.

**METHODS:**

This laboratory-based experimental study utilized human bronchial epithelial BEAS-2B cells exposed to cigarette smoke extract (CSE) and a mouse model of CS-induced emphysema. Molecular interventions included mutation of PRKN at Ser131, and knockdown of mitogen-activated protein kinase kinase 3 (MKK3) to modulate the downstream p38 mitogen-activated protein kinase (p38 MAPK) pathway. Mitophagy activity, mitochondrial reactive oxygen species (ROS), and senescence markers were evaluated.

**RESULTS:**

Exposure to cigarette smoke extract (CSE) increased MKK3 expression and activated the p38 MAPK pathway, leading to phosphorylation of PRKN at Ser131. This phosphorylation was accompanied by reduced mitophagy-related readouts and increased mitochondrial ROS. Both MKK3 knockdown and modulation of mitochondrial quality-control pathways were associated with improved mitophagy-related readouts and reduced senescence markers under CSE exposure. Moreover, cells expressing the PRKN S131A mutant exhibited significantly improved mitophagy flux, reduced ROS levels, and attenuated senescence compared to wild-type PRKN. *In vivo*, emphysematous lungs showed increased MKK3 and senescence markers alongside decreased PRKN and PTEN-induced kinase 1 (PINK1) expression.

**CONCLUSIONS:**

Our findings suggest that the MKK3/p38 MAPK–PRKN Ser131 axis contributes to CS-induced mitophagy impairment and epithelial senescence. Additional studies are needed to strengthen the evidence and evaluate the translational potential of targeting this pathway in COPD.

## INTRODUCTION

Chronic obstructive pulmonary disease (COPD) is a major global cause of mortality and disability, characterized by persistent respiratory symptoms and airflow limitation that is not fully reversible, most commonly driven by long-term exposure to noxious particles and gases such as cigarette smoke^1,2^. COPD typically develops from mid-life onward and its burden increases with aging, underscoring the importance of aging-related mechanisms in disease progression^1^.

Oxidative stress is a hallmark of COPD. Elevated oxidative stress biomarkers have been detected in airway-derived samples from patients with COPD, indicating a sustained oxidant burden in the diseased lung^3^. Experimentally, cigarette smoke/cigarette smoke extract (CS/CSE) increases mitochondrial reactive oxygen species (mtROS), disrupts mitochondrial homeostasis, and promotes airway epithelial senescence^4^. Because mitochondria are both a major source and a key target of ROS, persistent mtROS overload can amplify oxidative injury and downstream inflammatory signaling, including inflammasome-related pathways^5,6^. Consistent with this concept, prolonged smoke exposure alters mitochondrial structure and function in airway epithelial cells, and smoke stress promotes mitochondrial fragmentation that accompanies epithelial senescence^7,8^.

Mitophagy is essential for mitochondrial quality control, and the PINK1/PRKN pathway is a canonical mechanism for selectively eliminating damaged mitochondria^9-11^. Upon mitochondrial depolarization, PINK1 accumulates on the outer mitochondrial membrane and initiates a phosphorylation cascade in which PINK1 phosphorylates PRKN and ubiquitin to promote PRKN activation and ubiquitin-dependent signaling on damaged mitochondria^12,13^. Phosphorylated ubiquitin serves as a high-affinity PRKN receptor and drives conformational activation of PRKN, thereby amplifying mitochondrial ubiquitination and recruitment of mitophagy receptors^14-16^. Downstream, the PINK1–PRKN ubiquitylation program recruits OPTN/NDP52 and activates TBK1 to execute mitophagy^17^. Consistently, Ito et al.^18^ extracted lung homogenate from COPD patients and verified that the level of PRKN protein was positively associated with lung function of patients, which indicated PRKN may play a key regulatory role in the pathogenesis of COPD. Nevertheless, while cigarette smoke has been linked to impaired mitophagy and epithelial senescence, the upstream signaling events that functionally disable PRKN in COPD-relevant airway epithelial injury remain insufficiently defined^4,18^.

Mitogen-activated protein kinase kinase 3 (MKK3), an upstream activator of p38 MAPK, has been implicated in mitochondrial quality control and smoke-induced inflammation. Genetic loss of MKK3 improves mitochondrial function and mitochondrial quality control and attenuates inflammatory responses in experimental lung injury and cigarette smoke exposure^19-21^. Notably, p38 MAPK has been reported to phosphorylate PRKN at Ser131, weakening PRKN function and impairing mitophagy^22^. However, whether CSE activates an MKK3/p38-dependent PRKN Ser131 phosphorylation axis to causally suppress PRKN-mediated mitophagy, thereby driving mtROS accumulation and epithelial senescence, has not been established in COPD-relevant models.

Therefore, this study aimed to determine whether cigarette smoke extract (CSE) activates the MKK3/p38 MAPK pathway to induce PRKN Ser131 phosphorylation, thereby impairing PRKN-mediated mitophagy and promoting epithelial cell senescence. We further sought to clarify the underlying mechanism using MKK3 knockdown, assessment of p38 MAPK phosphorylation/activation, and the phosphorylation-deficient PRKN S131A mutant, and to validate these findings in a CS/LPS-induced emphysema mouse model.

## METHODS

### Study design and reporting

This laboratory-based experimental study included *in vitro* BEAS-2B cells exposed to 5% cigarette smoke extract (CSE) and an *in vivo* CS/LPS-induced emphysema mouse model (n=7/group). Primary outcomes included mitophagy-related readouts, mitochondrial ROS, and senescence markers assessed by immunofluorescence, Western blotting, RT-qPCR, and ELISA. The animal study is reported in accordance with the ARRIVE 2.0 guidelines (Supplementary file). All experiments were conducted between January 2021 and June 2024 in China.

### *In vivo* experiments: Animals and treatments

Male C57BL/6J mice (7 weeks old, 18–20 g) were obtained from the Animal Experimental Center of Qilu Hospital and housed at 22 ± 2 °C, 40–60% humidity, under a 12-h light/dark cycle, with ad libitum access to food and water. Mice were acclimatized for 7 days before experiments and randomly allocated (random number method) to room-air control or cigarette smoke (CS)-exposed groups (n=7/group). For COPD induction, mice received intranasal lipopolysaccharide (LPS; 30 μg) on days 2 and 29 and were exposed to smoke from 20 cigarettes/day, 5 days/week for 1 month in a 60×57×100 cm smoking chamber; room-air controls were handled identically without CS exposure. Animals were monitored daily for general condition and signs of distress, and humane endpoints were applied according to institutional guidelines. On day 30, mice were deeply anesthetized with sodium pentobarbital, blood was collected by cardiac puncture for serum preparation, and mice were euthanized by CO2 inhalation prior to tissue harvesting. Lung tissues and bronchoalveolar lavage fluid (BALF) were collected as described above. Outcome quantification and analysis were performed blinded to group allocation. No animals were excluded from the endpoint analyses; the final numbers analyzed were n=7 per group.

### *In vitro* experiments: Cell model

*Cell culture and CSE exposure*

Immortalized BEAS-2B cells (Procell Technology, Wuhan, China) within 40 passages were cultured in Dulbecco’s Modified Eagle Medium (DMEM) (Gibco) supplemented with 10% fetal bovine serum (FBS) (Biological Industries) at 37°C in 5% CO_2_. Cells were exposed to 5% cigarette smoke extract (CSE) for 24 h when indicated; cells cultured under identical conditions without CSE exposure served as the control group (Control/NC). Primary antibodies included β-actin (Beyotime, AF0003), glyceraldehyde-3-phosphate dehydrogenase (GAPDH) (Beyotime, AF5009), MEK3/MKK3 (Abcam, ab195037), p16 (Abcam, ab108349), p21 (CST, 2947), PINK1 (CST, 6946), Parkin (Abcam, ab77924), phospho-Parkin-Ser131 (Absci, 12371), LC3B (CST, 3868), and TOMM20 (Abcam, ab283317). Fluorescent secondary antibodies were from Beyotime or Abbkine. Mdivi-1 (GLPBIO) and Torin1 (Selleck) were used as mitophagy inhibitors and inducers, respectively.

*Preparation of CSE*

CSE was prepared as previously described. Mainstream smoke from one cigarette was bubbled into 10 mL of serum-free DMEM, filtered, and adjusted to pH 7.35–7.45. Absorbance at 320 nm was measured, and 100% CSE was defined as optical density at 320 nm (OD320) = 0.74 ± 0.05. CSE was diluted to working concentrations and used within 30 min.

*Molecular interventions: siRNA transfection*

Small interfering RNAs (siRNAs) (RiboBio) targeting MKK3 were transfected into BEAS-2B cells using Lipofectamine 2000 (Invitrogen). Knockdown efficiency was verified by RT-qPCR. The following siRNA sequences were used: negative control (si-NC, UUCUCCGAACGUGUCACGUTT) and MKK3-targeting siRNAs (si-MKK3-326, GAAGAAGGAUCUACGGAUATT; si-MKK3-612, GCACGGUCGACUGUUUCUATT; si-MKK3-994, GCUACAAUGUCAAGUCCGATT).

*Molecular interventions: lentiviral infection*

Lentiviral vectors overexpressing PRKN or PRKN-S131A (Genechem, Shanghai) were transduced into BEAS-2B cells. Stable lines were selected with puromycin and validated by fluorescence observation and by confirming PRKN expression at mRNA and protein levels.

### Outcome measurements/assays

*Western blotting*

Cells were lysed in radioimmunoprecipitation assay (RIPA) buffer (Beyotime). Equal amounts of protein (10–30 μg) were resolved by 10–15% sodium dodecyl sulfate–polyacrylamide gel electrophoresis (SDS-PAGE) and transferred to polyvinylidene difluoride (PVDF) membranes (0.45 μm). Membranes were incubated with primary antibodies overnight at 4°C, followed by horseradish peroxidase (HRP)-conjugated anti-rabbit or anti-mouse secondary antibodies (CST). Bands were visualized by chemiluminescence (Tanon 4800) and quantified using ImageJ, with normalization to GAPDH.

*Immunofluorescence*

Cells grown on slides and treated as indicated were fixed with 4% paraformaldehyde, permeabilized with 0.03% Triton X-100, and blocked with 5% bovine serum albumin (BSA). Primary antibodies were applied overnight at 4°C, followed by fluorescent secondary antibodies for 1 h at room temperature. Images were captured using an OLYMPUS IX81 microscope. LC3B and TOMM20 signals were merged to assess mitophagy.

*RT-qPCR*

Total RNA was isolated using TRIzol (Invitrogen) and reverse-transcribed with the PrimeScript RT kit (Takara). Reverse transcription quantitative polymerase chain reaction (RT-qPCR) was performed using TB Green Premix Ex Taq II (Takara). GAPDH served as an internal control. Relative mRNA expression was calculated using the 2^-ΔΔCt^ method.

*Water-soluble tetrazolium salt-1 (WST-1) cytotoxicity assay*

Cells (5×10^3^/well) were seeded into 96-well plates and incubated with CSE at indicated concentrations for 24–72 h. WST-1 solution was added and mixed, and absorbance at 450 nm was recorded to assess cell viability.

*ROS detection*

ROS levels were detected using a dihydroethidium (DHE)-based assay kit (Beyotime). After treatment, cells were incubated with DHE and imaged by fluorescence microscopy. Fluorescence intensity was quantified with ImageJ.

*Histology and immunohistochemistry*

Lung tissues were processed following standard procedures. Sections were incubated with primary rabbit antibodies and HRP-conjugated secondary antibody (CST, 7074). 3,3'-diaminobenzidine (DAB) substrate (BosterBio) was used for color development.

*Enzyme-linked immunosorbent assay (ELISA)*

Serum samples from control and emphysema mice were collected and centrifuged at 2500g for 10 min. IL-8, TNF-α, and IL-6 levels were determined using ELISA kits (Elabscience) according to the manufacturer’s instructions.

### Ethical approval

All animal procedures were reviewed and approved by the Scientific Research Ethics Committee of Qilu Hospital, Shandong University (Approval No. KYLL-2022(ZM)-1062; Date: 1 November 2022) and all experiments were performed in accordance with institutional guidelines for the care and use of laboratory animals. The *in vitro* experiments were conducted using the immortalized BEAS-2B cell line purchased from a commercial supplier; this study did not involve recruitment of human participants or collection of identifiable human specimens.

### Statistical analysis

Data were analyzed using SPSS 22.0 and GraphPad Prism 7.0. Results are presented as mean ± standard deviation (SD). Unless otherwise specified, *in vitro* experiments were performed with at least three independent biological replicates (n=3), and representative images are shown. Comparisons between two groups were performed using an unpaired two-tailed Student’s t-test. For comparisons among three or more groups, one-way analysis of variance (ANOVA) followed by a *post hoc* multiple-comparisons test was used, as indicated in the figure legends. Normality and homogeneity of variance were assessed prior to parametric testing; when assumptions were not met, appropriate non-parametric tests were used. Western blot grayscale values were quantified using ImageJ. Differences were considered statistically significant at p<0.05.

## RESULTS

### CSE increases MKK3 and is accompanied by altered mitophagy-related readouts and increased senescence markers in BEAS-2B cells

**Figure 1:**
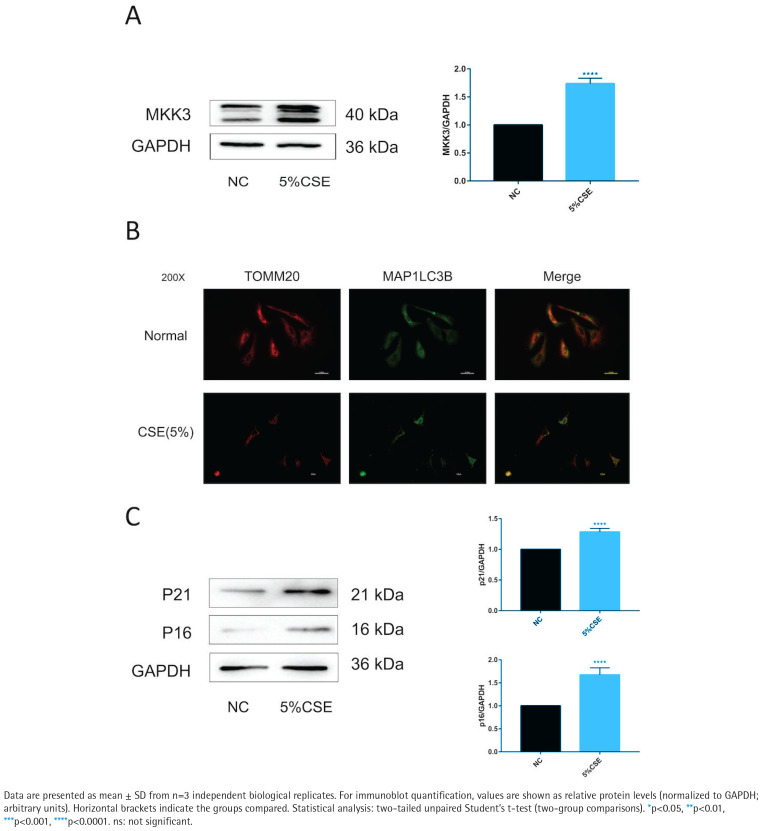
CSE exposure increases MKK3 and is accompanied by reduced TOMM20–MAP1LC3B colocalization and increased senescence markers in BEAS-2B cells (5% CSE, 24 h; n=3): A) Representative immunoblot of mitogen-activated protein kinase kinase 3 (MKK3) in BEAS-2B cells in the negative control (NC) and 5% cigarette smoke extract (CSE) groups, with densitometric quantification normalized to glyceraldehyde-3-phosphate dehydrogenase (GAPDH); B) Representative immunofluorescence images (200×) showing translocase of outer mitochondrial membrane 20 (TOMM20) and microtubule-associated proteins 1A/1B light chain 3B (MAP1LC3B/LC3B), and merged images, in the indicated groups; C) Representative immunoblots of cyclin-dependent kinase inhibitor 2A (p16/CDKN2A) and cyclin-dependent kinase inhibitor 1A (p21/CDKN1A) with densitometric quantification normalized to GAPDH

MKK3 protein levels were higher in CSE-treated BEAS-2B cells than in untreated controls (Figure 1A). TOMM20–MAP1LC3B colocalization was reduced under CSE exposure (Figure 1B). In parallel, p16 and p21 protein levels were increased in the CSE condition (Figure 1C). To support these imaging findings at the pathway level, PINK1 and PRKN readouts under the same CSE context are provided in Supplementary file Figure S1.

### MKK3 knockdown attenuates CSE-associated changes in mitophagy-related imaging readouts and reduces senescence markers across modulatory conditions

**Figure 2:**
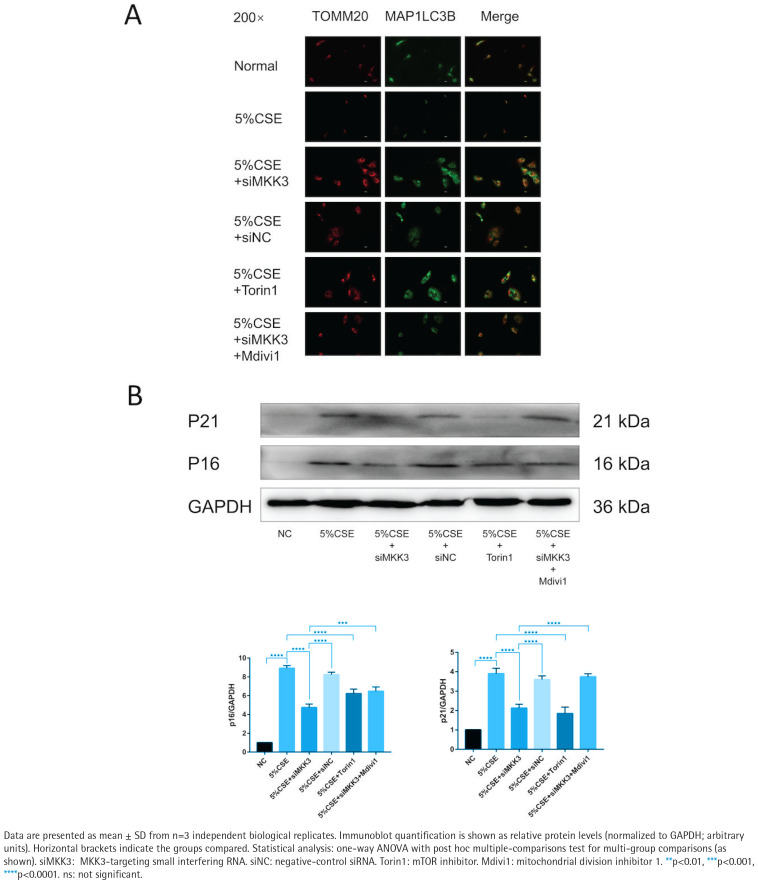
MKK3 knockdown is associated with improved TOMM20–MAP1LC3B colocalization and reduced senescence markers under CSE exposure with modulatory conditions (5% CSE, 24 h; n=3): A) Representative immunofluorescence images (200×) of TOMM20, MAP1LC3B (LC3B), and merged images in the following groups: Normal, 5% CSE, 5% CSE + siMKK3, 5% CSE + siNC, 5% CSE + Torin1, and 5% CSE + siMKK3 + Mdivi1; B) Representative immunoblots of p16 and p21 with densitometric quantification normalized to GAPDH across the indicated groups

Across the indicated treatment conditions, TOMM20–MAP1LC3B colocalization was higher in the siMKK3 condition than in the CSE condition (Figure 2A). Under the same experimental framework, p16 and p21 protein levels were lower in the siMKK3 condition than in the CSE condition (Figure 2B). The efficiency of MKK3 silencing and siRNA-related validation is shown in Supplementary file Figure S2. In addition, PINK1/PRKN molecular readouts across the corresponding conditions are provided in Supplementary file Figure S3.

### MKK3 knockdown is associated with reduced p38 MAPK phosphorylation and reduced PRKN Ser131 phosphorylation

**Figure 3:**
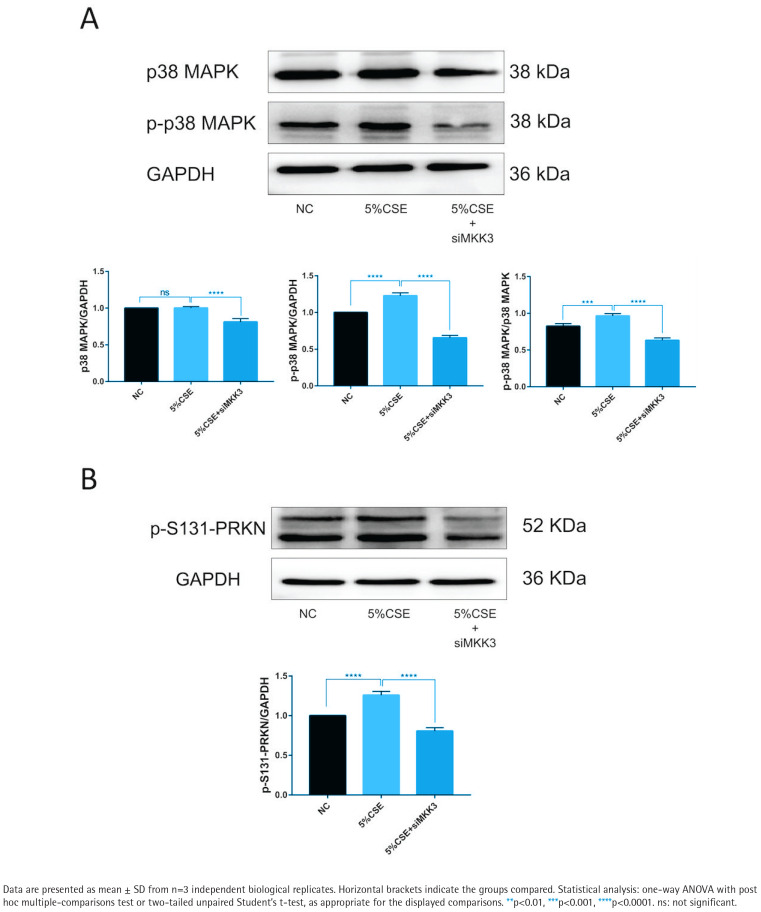
MKK3 knockdown is associated with reduced p38 MAPK phosphorylation and reduced PRKN Ser131 phosphorylation under CSE exposure (5% CSE, 24 h; n=3): A) Representative immunoblots and densitometric quantification of phosphorylated p38 MAPK (p-p38 MAPK) and total p38 MAPK in the indicated groups, with quantification shown as relative intensity normalized to GAPDH; B) Representative immunoblot and densitometric quantification of PRKN (Parkin) phosphorylated at Ser131 (p-S131-PRKN) in the indicated groups, normalized to GAPDH

p38 MAPK phosphorylation was increased under CSE exposure, whereas total p38 MAPK levels were comparatively stable (Figure 3A). p38 MAPK phosphorylation was lower in the siMKK3 condition than in the CSE condition (Figure 3A). PRKN Ser131 phosphorylation (p-S131-PRKN) was increased under CSE exposure and was reduced in the siMKK3 condition (Figure 3B). Additional supporting blots and related validation panels are included in the supplementary dataset as part of the figure set (see Supplementary file Figure S2 for knockdown validation and Supplementary file Figure S3 for pathway-level readouts under matched conditions).

### Emphysema mouse lungs show concordant changes in MKK3, PINK1/PRKN, and senescence markers

**Figure 4:**
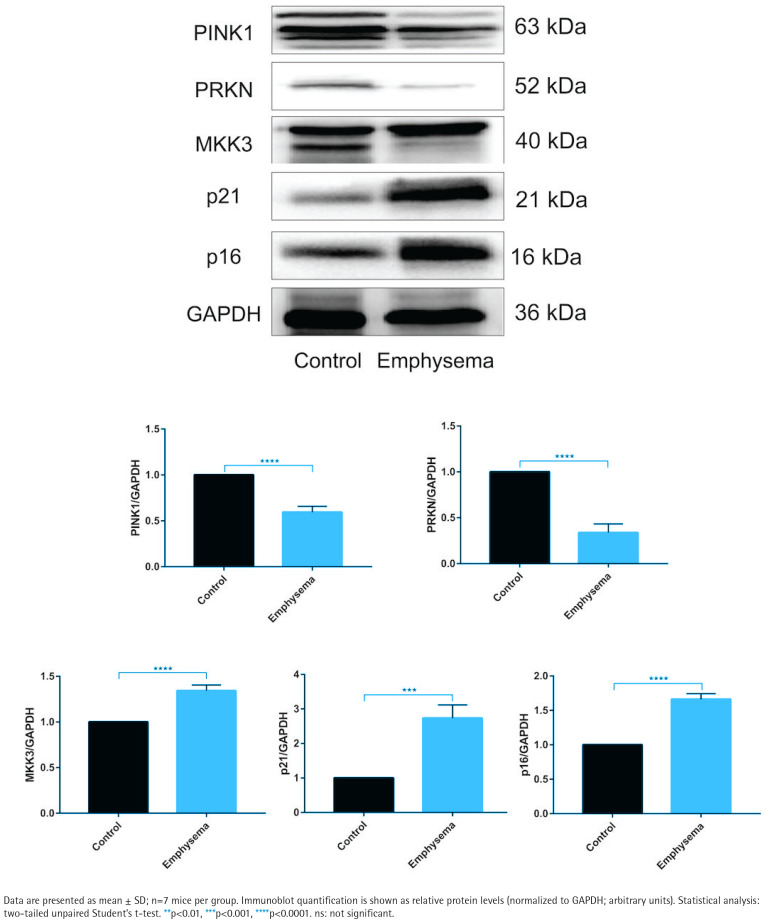
Emphysema mouse lungs show increased MKK3 and senescence markers and reduced PINK1/PRKN levels (20 cigarettes/day, 5 days/week, 1 month; n=7 mice/group): Representative immunoblots and densitometric quantification of PTEN-induced kinase 1 (PINK1), PRKN (Parkin), MKK3, p16 (CDKN2A), and p21 (CDKN1A) in lung tissues from control and emphysema mice, normalized to GAPDH

In emphysema mouse lungs, MKK3, p16, and p21 levels were higher than in controls, whereas PINK1 and PRKN levels were lower (Figure 4). Histological evidence and inflammatory-marker measurements supporting the emphysema model are provided in Supplementary file Figure S4.

### PRKN manipulation: construct validation and extended readouts under PRKN overexpression and PRKN S131A

PRKN overexpression and PRKN S131A expression are validated at the protein and mRNA levels in Supplementary file Figure S5. Under CSE exposure, TOMM20–MAP1LC3B colocalization and ROS-related imaging readouts under PRKN manipulation are provided in Supplementary file Figure S6, and senescence-marker measurements under extended conditions (including modulatory treatment) are shown in Supplementary file Figure S7.

## DISCUSSION

In this study, we identify MKK3 as a key factor associated with cigarette smoke-induced disruption of mitochondrial quality control in airway epithelial cells. CSE exposure increased MKK3 expression and was accompanied by enhanced p38 MAPK phosphorylation and PRKN Ser131 phosphorylation, together with reduced PRKN/PINK1-related mitophagy readouts and increased senescence markers. Moreover, suppression of the p38 MAPK pathway via MKK3 silencing attenuated these changes, and expression of the phosphorylation-deficient PRKN S131A mutant further supported the functional relevance of Ser131 phosphorylation. Below, we discuss these findings in the context of prior literature and their potential implications for COPD pathogenesis.

Clinical and experimental studies have demonstrated that COPD patients exhibit marked mitochondrial dysfunction^23,24^. Mitochondria are highly sensitive to oxidative stress; under physiological conditions, mitochondrial ROS act as second messengers to maintain intracellular homeostasis^25^. Excess ROS damages mitochondrial DNA (mtDNA)^26^, alters electron transport efficiency, and promotes further ROS accumulation, forming a vicious cycle. Persistent cigarette smoke exposure produces high ROS levels and leads to mitochondrial structural abnormalities, including loss of cristae, swelling, and fragmentation, consistent with observations in airway epithelial cells from COPD patients^27^. These changes are associated with decreased oxygen consumption, ATP production, mitochondrial membrane potential, and electron transport chain activity. Excess mtROS can further trigger inflammation via tumor necrosis factor receptor (TNFR), MAPK, and NLR family pyrin domain containing 3 (NLRP3) signaling pathways.

Mitophagy – particularly the PINK1/PRKN pathway – is a central mechanism of mitochondrial quality control. Loss of PINK1 or PRKN enhances inflammation, activates the cGAS–STING (cyclic GMP–AMP synthase–stimulator of interferon genes) pathway, and increases interleukin-1 beta (IL-1β) secretion^28^. Patients with PRKN/PINK1 mutations exhibit elevated interleukin-6 (IL-6) and circulating cell-free mtDNA^29^, highlighting the link between impaired mitophagy and inflammation-related diseases. Cellular senescence, another major COPD phenotype, is also driven by mitochondrial dysfunction^30^, telomere damage, and accumulated mtDNA mutations^31,32^. PRKN dysfunction accelerates aging phenotypes and shortens lifespan, whereas its overexpression exerts protective effects^33^. Previous studies associated CS exposure with impaired mitophagy and increased senescence in airway epithelial cells. Ito et al.^18^ showed that ROS accumulation impairs mitophagy and drives senescence, while Hara et al.^8^ demonstrated that PRKN is a rate-limiting factor in CS-induced mitophagy.

In this context, we confirmed that CSE increased MKK3 levels and impaired mitophagy in BEAS-2B cells, accompanied by reduced PINK1/PRKN proteins and decreased LC3 puncta. Senescence markers p16 and p21 were elevated, supporting that CSE induces mitochondrial dysfunction and senescence. Silencing MKK3 effectively restored mitophagy and alleviated senescence. Although the transfection reagent Lipofectamine 2000 increased LC3 fluorescence in si-NC cells, the fluorescence was diffuse with poor colocalization and did not reduce p16/p21 to the same extent as MKK3 knockdown. These findings indicate that the protective effects observed following MKK3 inhibition are independent of Lipo3000 and mediated through enhanced mitophagy. Rescue experiments using Mdivi1 further confirmed that restored mitophagy is required for the anti-senescence effects of MKK3 inhibition.

Mechanistically, MKK3 is known to activate p38 MAPK, a kinase involved in mitochondrial dysfunction. In Parkinson’s disease, α-synuclein A53T activates p38 MAPK, which phosphorylates PRKN at Ser131 and compromises its function. Consistent with this, our results showed that CSE-induced up-regulation of MKK3 increased p38 MAPK phosphorylation and PRKN Ser131 phosphorylation, while MKK3 knockdown reversed both. To validate the functional significance of Ser131, we introduced a PRKN S131A mutant via lentiviral vectors. PRKN S131A most effectively restored mitophagy, reduced mitochondrial ROS, and attenuated senescence compared with wild-type PRKN, confirming Ser131 as a critical damage site for MKK3-p38 signaling.

Recent studies have expanded the regulatory network of PRKN-dependent mitophagy. PRKN can be modulated via protein kinase A (PKA)-p38-cAMP response element-binding protein (CREB), deubiquitination by ubiquitin-specific protease 33 (USP33), and microRNA regulation such as miR-218^34^. PRKN-independent pathways (BNIP3L, BNIP3, FUNDC1, FKBP8, BCL2L13, AMBRA1, PHB2, cardiolipin, ceramide) further emphasize the complexity of mitochondrial quality control^35^.

*In vivo*, our mouse model with CS plus LPS reproduced emphysematous lesions and increased inflammatory cytokines. Lung tissues showed elevated MKK3, p16, and p21, with reduced PINK1/PRKN, indicating impaired mitophagy and intensified senescence. Although previous studies suggested that PINK1-/- mice are resistant to CS-induced emphysema, this discrepancy may reflect differences in modeling strategies or tissue contexts. Further validation using human COPD samples is warranted.

### Limitations

This study has several limitations. First, our conclusions are based on laboratory models, including BEAS-2B cells exposed to cigarette smoke extract and a CS/LPS-induced emphysema mouse model; therefore, the generalizability to human COPD requires further validation in clinical samples. Second, the *in vitro* system relies on a single immortalized bronchial epithelial cell line and an acute CSE exposure paradigm, which may not fully recapitulate the cellular heterogeneity, chronic exposure patterns, and microenvironmental interactions present in human airways. Third, while our data support involvement of the MKK3/p38 MAPK–PRKN Ser131 pathway in mitophagy impairment and senescence, additional studies using primary human airway epithelial cells and further *in vivo* validation will be important to strengthen the evidence base and translational relevance.

## CONCLUSIONS

Our findings support a model in which cigarette smoke exposure is associated with MKK3/p38 MAPK activation and PRKN Ser131 phosphorylation, accompanied by impaired PRKN-dependent mitophagy and increased epithelial senescence markers *in vitro* and *in vivo*. These findings highlight the MKK3–p38 MAPK–PRKN pathway as a mechanistic link between cigarette smoke exposure, mitophagy disruption, and epithelial senescence in COPD. Further studies, particularly in primary human samples and additional *in vivo* models, are needed to strengthen the evidence and define the translational relevance of this pathway.

## Supplementary Material



## Data Availability

The data supporting this research are available from the authors on reasonable request.
