# Supplementary material for: PRKN Ser131 phosphorylation promotes cigarette smoke-induced mitophagy impairment and epithelial cell senescence via MKK3/p38 MAPK activation: An in vitro and in vivo study
Source: Tob Induc Dis. 2026 May 27;24:10.18332/tid/218816. doi: 10.18332/tid/218816 (PMC13215348; doi:10.18332/tid/218816)
Supplement: Supplementary file 1 [file TID-24-74-s1.pdf]

Supplementary Files

-ARRIVE guidelines 2.0: author checklist
-Supplementary figures 1-7

NOTE: Please save this file locally before filling in the table, DO NOT work on the file within your internet browser as changes will not be saved. Adobe Acrobat Reader (available free here) is recommended for completion.

The ARRIVE guidelines 2.0: author checklist

The ARRIVE Essential 10

These items are the basic minimum to include in a manuscript. Without this information, readers and reviewers cannot assess the reliability of the findings.

Item		Recommendation	Section/line number, or reason for not reporting
**Study design**	1	For each experiment, provide brief details of study design including:	
		a. The groups being compared, including control groups. If no control group has been used, the rationale should be stated.	Page 2– 3, "In vivo experiments: Animals and treatments" (room-air control vs. CS-exposed group; n=7/group)
		b. The experimental unit (e.g. a single animal, litter, or cage of animals).	Cage of animals; Page 3, "In vivo experiments: Animals and treatments" (mice were housed and exposed as a group within a 60× 57× 100 cm smoking chamber)
**Sample size**	2	a. Specify the exact number of experimental units allocated to each group, and the total number in each experiment. Also indicate the total number of animals used.	Page 2, "Study design and reporting" (n=7/group); Page 3, "In vivo experiments: Animals and treatments"
		b. Explain how the sample size was decided. Provide details of any *a priori* sample size calculation, if done.	The sample size was determined based on previously published similar studies in the field; Page 2, "Study design and reporting"
**Inclusion and exclusion criteria**	3	a. Describe any criteria used for including and excluding animals (or experimental units) during the experiment, and data points during the analysis. Specify if these criteria were established *a priori*. If no criteria were set, state this explicitly.	Page 3, "In vivo experiments: Animals and treatments" (male C57BL/6J mice, 7 weeks old, 18– 20 g)
		b. For each experimental group, report any animals, experimental units or data points not included in the analysis and explain why. If there were no exclusions, state so.	Page 3, "In vivo experiments: Animals and treatments" (no animals were excluded)
		c. For each analysis, report the exact value of *n* in each experimental group.	Page 3, "In vivo experiments: Animals and treatments" (n=7 per group)
**Randomisation**	4	a. State whether randomisation was used to allocate experimental units to control and treatment groups. If done, provide the method used to generate the randomisation sequence.	Page 3, "In vivo experiments: Animals and treatments" (random number method)
		b. Describe the strategy used to minimise potential confounders such as the order of treatments and measurements, or animal/cage location. If confounders were not controlled, state this explicitly.	Page 3, "In vivo experiments: Animals and treatments"
**Blinding**	5	Describe who was aware of the group allocation at the different stages of the experiment (during the allocation, the conduct of the experiment, the outcome assessment, and the data analysis).	Page 3, "In vivo experiments: Animals and treatments" (outcome quantification and analysis performed blinded to group allocation)
**Outcome measures**	6	a. Clearly define all outcome measures assessed (e.g. cell death, molecular markers, or behavioural changes).	Page 2, "Study design and reporting" (mitophagy-related readouts, mitochondrial ROS, senescence markers)
		b. For hypothesis-testing studies, specify the primary outcome measure, i.e. the outcome measure that was used to determine the sample size.	senescence markers). No formal a priori sample size calculation was performed based on a primary outcome measure; sample size was determined based on previously published similar studies in the field (see also Item 2b).
**Statistical methods**	7	a. Provide details of the statistical methods used for each analysis, including software used.	Page 4, "Statistical analysis"
		b. Describe any methods used to assess whether the data met the assumptions of the statistical approach, and what was done if the assumptions were not met.	Page 4, "Statistical analysis" (normality and homogeneity of variance assessed prior to parametric testing)
**Experimental animals**	8	a. Provide species-appropriate details of the animals used, including species, strain and substrain, sex, age or developmental stage, and, if relevant, weight.	Page 2– 3, "In vivo experiments: Animals and treatments" (male C57BL/6J, 7 weeks, 18– 20 g)
		b. Provide further relevant information on the provenance of animals, health/immune status, genetic modification status, genotype, and any previous procedures.	Page 2– 3, "In vivo experiments: Animals and treatments"; housing conditions described (22 ± 2° C, 40– 60% humidity, 12-h light/dark cycle)
**Experimental procedures**	9	For each experimental group, including controls, describe the procedures in enough detail to allow others to replicate them, including:	Page 3, "In vivo experiments: Animals and treatments" (what, how, when, where, why all described)
		a. What was done, how it was done and what was used.	LPS 30 μ g on days 2 and 29, and were exposed to mainstream smoke from 20 cigarettes/day in a 60× 57× 100 cm smoking chamber; room-air controls were handled identically without CS exposure)
		b. When and how often.	
		c. Where (including detail of any acclimatisation periods).	d treatments" (housed at 22 ± 2° C, 40– 60% humidity, 12-h light/dark cycle, with ad libitum access to food and water; acclimatised for 7 days before experiments)
		d. Why (provide rationale for procedures).	treatments" (CS/LPS-induced emphysema mouse model was used to reproduce key pathological features of COPD in vivo and to validate in vitro findings in a disease-relevant context)
**Results**	10	For each experiment conducted, including independent replications, report:	Pages 5– 8, Figure legends (mean ± SD, n=3 or n=7 per group as indicated)
		a. Summary/descriptive statistics for each experimental group, with a measure of variability where applicable (e.g. mean and SD, or median and range).	
		b. If applicable, the effect size with a confidence interval.	Not reported; statistical significance assessed using t-test/ANOVA with P<0.05; Page 4, "Statistical analysis"

Supplementary Figures

Supplementary Figure S1. CSE-associated changes in MKK3 and PINK1/PRKN readouts in BEAS-2B cells (5% CSE, 24 h; n=3).

(A) MKK3 mRNA levels in NC and 5% CSE groups.

(B) Representative immunoblots and densitometric quantification of PINK1 and PRKN protein levels normalized to GAPDH in NC and 5% CSE groups.

(C) PINK1 and PRKN mRNA levels in NC and 5% CSE groups.

Notes: qPCR is presented as relative mRNA expression ($2^{-\Delta\Delta Ct}$, fold of NC). Immunoblot quantification is presented as relative protein levels (normalized to GAPDH; arbitrary units). Data are mean ± SD; n=3. Statistical analysis: two-tailed unpaired Student's t-test. Significance notation as in main figures.

Supplementary Figure S2. Validation of MKK3 siRNA knockdown efficiency in BEAS-2B cells (n=3).

(A) MKK3 mRNA levels following transfection with the indicated MKK3-targeting siRNAs and controls (si326, si612, si994, siNC); siGAPDH is shown as a positive control.

(B) Representative immunoblots of MKK3 with GAPDH and β-tubulin loading controls and densitometric quantification.

Notes: Data are mean ± SD; n=3. qPCR and immunoblot quantification are defined as in Supplementary Fig. S1. Statistical analysis and significance notation as in main figures.

Supplementary Figure S3. PINK1/PRKN protein and mRNA readouts across siMKK3 and pharmacologic modulation conditions (5% CSE, 24 h; n=3).

(A) Representative immunoblots and densitometric quantification of PINK1 and PRKN protein levels normalized to GAPDH across the indicated groups (NC, 5% CSE, 5% CSE+siMKK3, 5% CSE+siNC, 5% CSE+Torin1, 5% CSE+siMKK3+Mdivi1).

(B) PINK1 and PRKN mRNA levels across the same groups.

Notes: qPCR and immunoblot quantification are defined as in Supplementary Fig. S1. Data are mean ± SD; n=3. Statistical analysis: one-way ANOVA with post-hoc multiple-comparisons test. Significance notation as in main figures.

Supplementary Figure S4. Histological and inflammatory characterization of the emphysema mouse model (1 month exposure; n=7 mice/group).

(A) Representative hematoxylin and eosin (H&E) staining of lung sections from control and CS+LPS groups (10× and 20×).

(B) ELISA measurements of inflammatory cytokines in the indicated groups.

Notes: Cytokine concentrations are presented in pg/mL (as labeled on axes). Data are mean ± SD; n=7 mice/group. Statistical analysis and significance notation as in main figures.
Abbreviations: CS, cigarette smoke; LPS, lipopolysaccharide; ELISA, enzyme-linked

immunosorbent assay.

Supplementary Figure S5. Validation of PRKN overexpression and PRKN S131A expression in BEAS-2B cells (n=3).
(A) Representative immunoblot and densitometric quantification of PRKN protein levels in NC, PRKN overexpression (OE), and PRKN S131A (Ser131→Ala) groups, normalized to GAPDH.
(B) PRKN mRNA levels in the indicated groups.
Notes: Data are mean ± SD; n=3. qPCR and immunoblot quantification are defined as in Supplementary Fig. S1. Statistical analysis and significance notation as in main figures.

Supplementary Figure S6. Mitophagy-related imaging readouts and ROS-related fluorescence under PRKN manipulation (5% CSE, 24 h; n=3).
(A) Representative immunofluorescence images (200×) of TOMM20, MAP1LC3B (LC3B), and merged images in the indicated groups (Normal, 5% CSE, 5% CSE+NC, 5% CSE+OE, 5% CSE+S131A).
(B) Representative fluorescence images of dihydroethidium (DHE) signal in the same groups.
Notes: Representative images are shown from n=3 independent experiments.
Abbreviations: DHE, dihydroethidium; ROS, reactive oxygen species.

Supplementary Figure S7. Senescence markers under PRKN overexpression/S131A with modulatory treatment (5% CSE, 24 h; n=3).
Representative immunoblots and densitometric quantification of p16 and p21 normalized to GAPDH in the indicated groups (Normal, 5% CSE, 5% CSE+NC, 5% CSE+OE, 5% CSE+S131A, 5% CSE+S131A+Mdivi1).
Notes: Data are mean ± SD; n=3. Immunoblot quantification is shown as relative protein levels (normalized to GAPDH; arbitrary units). Statistical analysis: one-way ANOVA with post-hoc multiple-comparisons test. Abbreviations: Mdivi1, mitochondrial division inhibitor 1. Significance notation as in main figures.

© 2026 Hu Y. et al.
